# Supplementary material for: Perceived needs of patients and family caregivers regarding home-based enteral nutritional therapy in South Africa: A qualitative study
Source: PLoS One. 2020 Feb 12;15(2):e0228924. doi: 10.1371/journal.pone.0228924 (PMC7015406; doi:10.1371/journal.pone.0228924)
Supplement: S2 Appendix — (DOCX) [file pone.0228924.s002.docx]

**SUPPORTING INFORMATION 2**: INTERVIEW GUIDE: FAMILY CARE GIVER

Site code: Date:

Start:

End:

**Demographics**

Age:

Gender:

Employment status:

Source of income:

**INTRODUCTION**

Good day Sir/Madam

Thank you for agreeing to be interviewed for my study. As you know, I got your contact details from hospital X as a family caregiver for Mr/Ms X. I am interested to know your perspective regarding your relative’s feeding method. The information you are going to share will contribute towards improving implementation of the national guidelines for people feeding through the tube outside hospital and in their homes. The aim of the guidelines is to ensure that everybody who qualifies, regardless of their location, receive quality nutritional care they deserve. Improved nutritional care may improve nutritional status, reduce complication and readmission rates and save health care costs for both patients and the state.

The interview will be recorded to make it easy to make interpretations later as I may not be able to remember everything you are going to say. Agreeing to participate means you are also agreeing to the recording. I would like to assure you that all the information you are going to share will be kept in strict confidence and will be used specifically for research purposes and nothing else. It will not be divulged even to nurses and doctors unless you give us permission to do so. Please make yourself comfortable and feel free.

The button is pressed, the interview begins:

1. **As somebody assisting X with their feeding Please tell me about how X is feeding and how long have they been feeding like this.**

- What is X eating and how?
- What were the reasons for this choice of feeding?
- Briefly describe how the decision for the choice of feeding was taken.
- Tell me how the feeding started and the education you were given on discharge as the family caregiver.

**2 Please describe the support you get regarding to your feeding**

- Where do you get the food products and material you are using and how you get the supply?

**3 How has the type of feeding affected your daily life with this type of feeding?**

- Can you do now what you used to do before you fell ill?

**4 Are you satisfied with the whole situation regarding X’s feeding?**

**5 Is there anything you would like to see done differently to improve feeding individuals in similar situations?**

Thank you for participating in my study.
