# Supplementary material for: Perceived needs of patients and family caregivers regarding home-based enteral nutritional therapy in South Africa: A qualitative study
Source: PLoS One. 2020 Feb 12;15(2):e0228924. doi: 10.1371/journal.pone.0228924 (PMC7015406; doi:10.1371/journal.pone.0228924)
Supplement: Attachment — Submitted filename: PONE RESPONSE TO REVIEWERS.docx [file pone.0228924.s003.docx]

RESPONSE TO REVIEWERS

| Comments from Reviewers | AUTHOR RESPONSE |
| --- | --- |
| Reviewer #2:  Very nice and well done qualitative study. Integration with Maslow's hierarchy is an excellent approach. I have only a few questions and comments. | Thank you for the opportunity to revise my paper, your comments made a lot of sense contributed a great deal in shaping my paper. |
| 1) Was intercoder/interrater reliability (kappa score) calculated, since there was a primary and co-coder? | No, it was deemed unnecessary since the co-coders can bring varied but valid perspectives to identify unique codes and themes in data. Additionally, we were not for generalizability of findings beyond the sample to the entire population as we had a small sample size. |
| 2) Do you have data regarding the total population of HEN patients in the district or community from which your purposive sample was identified? If so, please provide. | No, we do not have data regarding the total population of HEN patients as it has been mentioned in the study setting section that the district under study was referring HEN candidates to tertiary institutions in other districts. It was therefore difficult to keep record. |
| 3) Theme 1: Socioeconomic support needs, line 202 -- do you mean "was not sufficient" or "was insufficient". Incorrect to say "was not insufficient". | We meant was insufficient, thank you for pointing that out. |
| For your information, you may find the following paper and resource helpful in your work. Thompson CW, Durrant L, Barusch A, Olson L. Fostering coping skills and resilience in home enteral nutrition (HEN) consumer. Nutr Clin Pract 2006;21(6):557-565 <http://www.copingwell.com/copingwell/HENCopingManual.pdf> | Thank you for the information, it was helpful. |
| Reviewer #1:  This is a good article, but reading extremely difficult, I had to read paragraphs multiple times to understand what you mean, I suggest given to a colleague with a fresh eye for a good rewrite  2 = The definition for critical care = care of a patient in a life-threatening situation of an illness. Includes artificial life support system. = You need to remove the word critical illness / care from your article, the patients were not in ICU. You can say chronically ill / post ICU care . CHECK THE WHOLE ARTICLE FOR THIS  4 = rewrite = long to recover fully due to vulnerability to malnutrition and risk for infections.  20 = EN [already used before. do not write out again]  54 = Malsow mentioned, add Figure 1 after this paragraph, not only line 84  60 =add 'level 1'' in brackets  67 & 94 =HEN 89 = reword = doesn't make sense  92= [patient who had suffered one or more incidents of organ failure including the gastrointestinal tract and needed the support of others to meet their nutritional needs]... = GIT failure will mean no functioning GIT and TPN, so reword this. Patients are fed with PEG or HEN due to inability to feed themselves / cannot swallow etc = REWORD paragraph  98 = recruited = add in brackets [dietitian /stoma sister]  101-3 = CHANGE sentence = The dietician or stoma therapy nurse acted as recruiters in this study by providing the research information/documentation to prospective participants   125 [good rapport and to win the trust] = is this not the same thing? Reword  126 = verbal or written consent?   138 = DELETE [which was uploaded into the computer software program].  142-147 = REWRITE this, it is very confusing, I am still not sure how you analysed the data [A co-coder was engaged in coding the relevant information in the data and codes were sorted into categories in an attempt to answer the research questions. Analysis continued through creating themes to address the research question. Electronic files for each theme were then created and labelled, allowing for ease of access and management of data. Discussion and interpretation of the findings commenced thereafter]. Did you use any statistical software program or was it done manually? | Comment noted and a language editor has been consulted.  The definition for critical care = care of a patient in a life-threatening situation of an illness. Includes artificial life support system. = You need to remove the word critical illness / care from your article, the patients were not in ICU. You can say chronically ill / post ICU care . The whole article has been checked for this  Statement re-written as suggested.  Enteral nutrition written as EN.  Figure 1 added after the paragraph as recommended.  'level 1' added in brackets after pyramid.  HEN used in lines 67 and 94 which are  Re-worded to “a patient fed with PEG or HEN due to inability to swallow or feed themselves and needed the support of others to meet their nutritional needs”.  “dietitian or stoma therapy nurse” added in brackets.  Sentence changed to “The dietician or stoma therapy nurse acted as recruiters in this study by providing the research information/documentation to prospective participants”  It is, if you have good rapport with someone, you two work with trust and sympathy, it was an error and the latter has been removed, thank you.  Written consent.  Deleted.  Statement has been rewritten to avoid confusion. |
| Please read the following comments per line as in the PDF version I received: | Corrections were done per line as in the PDF we downloaded |
| It need language editing, some sentences very long and some start with ''OF''. | Language editor consulted |
| You are not allowed to mention product names or retailers in your article, it is against the Code. | That has been corrected and thank you for the comment. |
| References are only at the end of sentences, do they say all exactly the same? | Efforts have been made to avoid putting all references at the end of statements as is if they were saying exactly the same thing; 273-276 and 291-294. |
| If you defined abbreviation, used it in the rest of the article, do no write full words again. | Comment noted and the abbreviation issue has been corrected throughout the document. |
| 89 = reword = doesn't make sense | Reworded. |
| 92= [patient who had suffered one or more incidents of organ failure including the gastrointestinal tract and needed the support of others to meet their nutritional needs]... = GIT failure will mean no functioning GIT and TPN, so reword this. Patients are fed with PEG or HEN due to inability to feed themselves / cannot swallow etc = REWORD paragraph | Statement reworded and thank you. |
| 98 = recruited = add in brackets [dietitian /stoma sister] | Added |
| 101-3 = CHANGE sentence = The dietician or stoma therapy nurse acted as recruiters in this study by providing the research information/documentation to prospective participants | Sentence changed |
| 106 - 111 = MUST move to RESULTS: [Sixteen potential participants | Moved to results section |
| 113-114 = REMOVE sentence [ After obtaining ethical clearance and approval for the study from the University and the provincial Department of Health] | Removed |
| 114 = why only 3 months for data collection please explain. | It was due to time constraints on the researcher’s side. |
| 125 [good rapport and to win the trust] = is this not the same thing? Reword | Reworded and thank you. |
| 124, 12 = You mention ''One researcher'' and now ''The researcher'' did you use only one or more? Please check and change. | There was one researcher, the first author and a project supervisor, the co-author, it checked and changed throughout the study. |
| 138 = DELETE [which was uploaded into the computer software program]. | Deleted |
| 140 = ''celaining of data'' =please explain what you mean with this | This was referring to manually and informally identifying and removing errors, incomplete words and statements from the transcripts before analysis. There were no professional tools or software used for that where we can trace the steps, as such, it has been removed if it mentioning it leaves the reader with high expectations. |
| 142 = ço-coder'' = please define / explain | This was meant to be the co-author who was involved to confirm findings. |
| 142-147 = REWRITE this, it is very confusing, I am still not sure how you analysed the data [A co-coder was engaged in coding the relevant information in the data and codes were sorted into categories in an attempt to answer the research questions. Analysis continued through creating themes to address the research question. Electronic files for each theme were then created and labelled, allowing for ease of access and management of data. | The first author conducted the initial analysis and coding and the second author confirmed the findings. The section has been rewritten to make sense. |
| 164-5 = did you use one or more researcher? Please explain why if you did or didn't | One researcher, the first author collected data and second author (research supervisor) confirmed data analysis findings. |
| 188 = insert line 106-111 here | Line 106-111 inserted in the results section. |
| 208 = PLEASE explain what you mean with this [ The need for infrastructure for continuity of care was expressed by P1, P4 and P5] | The needed facilities for follow-up care either than tertiary hospitals where their PEGs were inserted. |
| 210-2 = remove commercial names of products and retailers [Glucerna, Diaben ,Nespray, Clicks] | [Glucerna, Diaben ,Nespray, Clicks replaced with diabetic formula, XXXX feed product and YYYY store. |
| 259, 280 = I suggest you contact some other provinces Dep of Health, because in gauteng they do provide supplements at Clinics if patient qualify according to TTO guidelines and there are some home-visits groups. | Comment noted and appreciated, but we feel it is a bit late for our study at this stage. However, it would be useful if we could include in our discussion section that other provinces in South Africa do provide the BTF supplements in the PHC clinics, unfortunately we could not locate any published material on that. |
| 270 = BTF = please define | BTF stated in full as Blenderised tube feeding, BTF in brackets. |
| 340 = Please explain on what basis, you say your study was representative of study group. Can you honesty say 7 is enough? Also explain here why the study was not extended after the 3 months when you were only left with 7. This is for me a limitation | Representativeness was referring to characteristics of the individuals that were required to address the research question than adequacy of the sample size. However, to avoid giving our readers an impression that we meant that 7 was enough to represent the entire population, the statement has been rephrased and stated as more of a limitation. |
