# Supplementary material for: Perceived needs of patients and family caregivers regarding home-based enteral nutritional therapy in South Africa: A qualitative study
Source: PLoS One. 2020 Feb 12;15(2):e0228924. doi: 10.1371/journal.pone.0228924 (PMC7015406; doi:10.1371/journal.pone.0228924)
Supplement: Attachment — Submitted filename: RESPONSE TO REVIEWERS.docx [file pone.0228924.s004.docx]

RESPONSE TO REVIEWERS

| Comments from Reviewers | AUTHOR RESPONSE |
| --- | --- |
| Reviewer #1: You did good work in addressing all the questions and shaping this paper! It read easy and you now understand fully what your goal was and your results. I think this is now a very good paper and you can be proud! Well done. | I thank the reviewers for their constructive feedback and comments, they added value to my work. |
| Reviewer #2: Most of the comments have been satisfactorily addressed. Even though you had a small sample, it is acceptable for a qualitative study in which the aim is to explore and describe perspectives ... and gain deeper understanding of phenomena under study; however, qualitative papers are enhanced by establishing inter-rater reliability, whether or not generalizability is limited. | Thank you for the comment and it is noted for future purposes. |
| Please add a statement in the limitation section (lines 324) that no inter-rater reliability was calculated however there was good agreement in coding and establishing representative themes between the researcher and second author. I understand the difficulty in establishing population data given the lack of infrastructure in the district, but it is difficult for the reader to appreciate the importance of need for HEN patients without some sense of incidence/prevalence of condition. | Statement added and thank you. |
| Could you explore whether data are available for # of patients discharged with HEN in the KwaZulu-Nata Province or any of the tertiary referral hospitals? IF none, please add a statement in the limitation section that there are no data on incidence/prevalence of HEN. | There are no data on incidence/prevalence of HEN in adults in the province or any of the tertiary institutions and that has been added in the limitation section . |
| In the conclusion, please address the need for education of primary care professionals in the district where patients are returning home, perhaps provided by professionals at the tertiary sites. | Thank you for the comment, a statement is added to the conclusion section. |
| The following sentences were unclear and may need rewriting: Line 7 - change nutritional risk to nutritionally at risk.  Lines 178-180, lines 197-198, lines 273-276. | Line 7 changed to nutritionally at risk.  Lines 178-180, 197-198 and 273-276 rewritten for clarity. |
| Consider editing the verbatim quotes by eliminating expressions (yhoo!) (man) etc and inserting clarifying words when missing. You can add a statement in the methods section that says something like verbatim quotes were edited for clarity and ease of reading by using ... for elimination of words/phrases or insertion of [ ] to represent added words. | Expressions eliminated and a statement has been added in the methodology section. |
| Would also clarify what is PEGI (page 10). | Corrected to PEG, which has been written in full in its initial mention. |
